# 

**Published:** 1888-07

**Authors:** 


WHOLE NUMBER CCCXXIII JULY, 1888. NUMBER 12. VOLUME XXVII.

LIBRA[illegible] INDE[illegible] S.G.

### THE BUFFALO Medical AND Surgical Journal

ESTABLISHED IN YEAR 1844.

**EDITOR:**

**THOS. LOTHROP, M. D.**

**ASSOCIATE EDITORS:**

FLOYD S. CREGO, M. D. CARLTON C. FREDERICK, M. D.

FRED. R. CAMPBELL, M. D. FRANK H. POTTER, M. D.

EDWARD B. ANGELL, M. D., Rochester. N. Y.

Entered at the Post-Office at Buffalo, N. Y., as Second-class Mail Matter.

